# Supplementary material for: Content-rich biological network constructed by mining PubMed abstracts
Source: BMC Bioinformatics. 2004 Oct 8;5:147. doi: 10.1186/1471-2105-5-147 (PMC528731; doi:10.1186/1471-2105-5-147)
Supplement: Additional File 2 — The original results of the above study (non-essential files are deleted to keep the file size under the limit set by BMC bioinformatics). [file 1471-2105-5-147-S2.bz2 › chilibotAdditionalFile2/dip40/747ID9150216E18129/html/EXBB_TONB.html]

 


 **EXBB** and **TONB** 
  
Found 83 abstracts in PubMed, retrieved 40.  
 

 What does Google say? 
 PDF only 
| .edu only 

---

**Interactive relationship** (e.g. stimulation, inhibition, etc)

**Neutral relationship**- The amino terminal signal anchor of  **TonB**  and its interaction with the cytoplasmic membrane protein  **ExbB**  are essential to this process.  Ref: 11087740 J Biol Chem, 2001
- The cytoplasmic membrane proteins  **ExbB**  and ExbD support  **TonB**  dependent active transport of iron siderophores and vitamin B12 across the essentially unenergized outer membrane of Escherichia coli.  Ref: 9811664 J Bacteriol, 1998
- The  **tonB**  Bb gene was situated upstream of  **exbB**  and exbD homologues and downstream of a putative Fur regulated promoter.  Ref: 10517598 Microbiology, 1999
- **ExbB**  and ExbD proteins are part of the  **TonB**  dependent energy transduction system and are encoded by the exb operon in Escherichia coli.  Ref: 12193634 J Bacteriol, 2002
- Both  **ExbB**  and ExbD were also required for association of  **TonB**  with the cytoplasmic membrane.  Ref: 12193634 J Bacteriol, 2002
- A second  **tonB**  gene in Pseudomonas aeruginosa is linked to the  **exbB**  and exbD genes.  Ref: 10689178 FEMS Microbiol Lett, 2000
- TetA  **TonB**  protein, which cannot interact with  **ExbB**  D, was likewise found associated with the outer membrane.  Ref: 9159515 Mol Microbiol, 1997
- These results indicated that the role of  **ExbB**  D in energy transduction is to bring  **TonB**  that has reached the outer membrane back to associate with the cytoplasmic membrane.  Ref: 9159515 Mol Microbiol, 1997
- Conserved residues Ser 16 and His 20 and their relative positioning are essential for  **TonB**  activity, cross linking of  **TonB**  with  **ExbB** , and the ability of  **TonB**  to respond to proton motive force.  Ref: 11087740 J Biol Chem, 2001
- Deletion of Trp 11 resulted in a  **TonB**  that remained active yet was unable to cross link with  **ExbB** .  Ref: 11087740 J Biol Chem, 2001
- Specifically, both  **ExbB**  and ExbD were found to be required for  **TonB**  to conformationally respond to proton motive force.  Ref: 12193634 J Bacteriol, 2002
- This genetic organization has been reported in Haemophilus influenzae, H. ducreyi, Pseudomonas putida and Vibrio cholerae, and may represent a separate lineage of evolution from that of the Enterobacteriaceae in which  **tonB**  is unlinked with the accessory genes  **exbB**  and exbD.  Ref: 9074497 Gene, 1997
- Interactions in the  **TonB**  dependent energy transduction complex  **ExbB**  and ExbD form homomultimers.  Ref: 9811664 J Bacteriol, 1998
- Energy for active transport is obtained from the proton motive force of the inner membrane through physical interaction with  **TonB**   **ExbB**  ExbD, an inner membrane complex.  Ref: 9886293 Nat Struct Biol, 1999
- The aim of this review is to describe an in vivo assay of the interactions taking place in the Tol Pal or  **TonB**   **ExbB**  ExbD envelope complexes in the periplasm of Escherichia coli and between them and colicins or g3p protein of filamentous bacteriophages.  Ref: 12423784 Biochimie< MedlineTA>Biochimie, 1999
- Because Trp 11 was demonstrably not involved in the actual cross linking, these results suggest that the  **TonB**   **ExbB**  interaction detected by cross linking occurred at a step in the energy transduction cycle distinct from the coupling of  **TonB**  to the electrochemical gradient.  Ref: 11087740 J Biol Chem, 2001
- Each suppressor  **ExbB**  restored at least partial function to the  **TonB**  mutants, although  **TonB**  delta V17, for which both the conserved motif and the register of the predicted transmembrane domain alpha helix are affected, was the most refractory.  Ref: 10209752 Mol Microbiol, 1999
- Here, two new mutations of the conserved  **TonB**  transmembrane domain SHLS motif were isolated,  **TonB**  S16L and H20Y, as were two new suppressors,  **ExbB**  V35E and V36D.  Ref: 10209752 Mol Microbiol, 1999
- This second defect was evident only after suppression by the  **ExbB**  mutants, which allow transmembrane domain mutants to be energized, and presented as the rapid turnover of  **TonB**  delta V17.  Ref: 10209752 Mol Microbiol, 1999

**Non-interactive relationship** (e.g. studied together, co-existance, homology, etc.)

- In addition, we found that transport across the outer membrane via FhuA,  **TonB** ,  **ExbB** , D rather than transport across the cytoplasmic membrane via FhuC, D, B was rate limiting.  Ref: 9613584 Mol Gen Genet, 1998
- Previous studies of a  **TonB**  transmembrane domain mutant  **TonB**  delta V17 and its phenotypical suppressor  **ExbB**  A39E suggested that  **TonB**  is conformationally sensitive.  Ref: 10209752 Mol Microbiol, 1999
- In this study, we report the per cell numbers of  **TonB** ,  **ExbB** , ExbD and FepA for cells grown under iron replete and iron limited conditions.  Ref: 11967085 Mol Microbiol, 2002
- Cloning and functional characterization of Neisseria gonorrhoeae  **tonB** ,  **exbB**  and exbD genes.  Ref: 9140974 Mol Microbiol, 1997
- The  **tonB** ,  **exbB**  and exbD1 genes of Xanthomonas campestris pv.  Ref: 10832632 Microbiology, 2000
- Cloning and characterization of the  **exbB**  exbD  **tonB**  locus of Pasteurella haemolytica A1.  Ref: 9074497 Gene, 1997
- Due to the low iron concentration in the plant tissue, the titre of viable bacteria of the ferric iron uptake mutants  **tonB** ,  **exbB**  and exbD1 decreased after leaf infiltration of pepper.  Ref: 10832632 Microbiology, 2000
- Thus, the sequence comparison identified the ORF1, ORF2 and ORF3 as gonococcal homologues of the E. coli  **tonB** ,  **exbB**  and exbD genes.  Ref: 9140974 Mol Microbiol, 1997
- Transport of bound chelates across the outer membrane depends upon  **TonB**   **ExbB**  ExbD, a cytoplasmic membrane localized complex that transduces energy from the proton motive force to high affinity receptors in the outer membrane.  Ref: 9643536 Mol Microbiol, 1998
- Results of insertional mutations, DNA and protein sequence analyses, phage sensitivity tests, transfection tests, complementation tests, and phage adsorption assays together with the cellular location of the proteins indicate that  **TonB** ,  **ExbB** , and ExbD1 are essential for penetration of phage phi L7.  Ref: 12646254 Biochem Biophys Res Commun, 2003
- Both full length  **TonB**  and truncated  **TonB**  consisting of the amino terminal two thirds achieved identical conformations in the cytoplasmic membrane, as determined by their abilities to cross link to the cytoplasmic membrane protein  **ExbB**  and their abilities to respond conformationally to the presence or absence of proton motive force.  Ref: 12823822 Mol Microbiol, 2003
- A comparison of the DNA flanking the  **exbB**  exbD  **tonB**  locus in P. haemolytica A1 and H. influenzae showed that the flanking regions are completely different between the two organisms.  Ref: 9074497 Gene, 1997
- Following incubation on media containing hemoglobin, N. meningitidis IR3436  **tonB**   **exbB**  exbD deletion mutant and N. gonorrhoeae PD3401  **tonB**  insertional mutant give rise to colonies which can grow with hemoglobin.  Ref: 10986265 J Bacteriol, 2000
- The reconstitution in Escherichia coli of the S. marcescens Has system demonstrated that haem uptake is dependent on the E. coli  **ExbB** , ExbD and  **TonB**  proteins.  Ref: 11737642 Mol Microbiol, 2001
- Cross linking analysis of epitope tagged  **ExbB**  and ExbD proteins with  **TonB**  antisera afforded detection of a previously unrecognized  **TonB**  ExbD cross linked complex and confirmed the composition of the  **TonB**   **ExbB**  cross linked complex.  Ref: 9811664 J Bacteriol, 1998
- A putative Fur binding sequence located upstream from  **tonB**  was detected in a Fur titration assay, indicating that the  **tonB**   **exbB**  exbD operon may be Fur repressed in high iron growth conditions.  Ref: 10722583 Infect Immun, 2000
- A complex of three membrane spanning proteins  **TonB** ,  **ExbB**  and ExbD couples the chemiosmotic potential of the cytoplasmic membrane with siderophore uptake across the outer membrane.  Ref: 12409204 Biochim Biophys Acta, 2002
- By mutational analysis, the  **tonB** ,  **exbB** , and exbD1 genes were shown to be essential for ferric iron import in X. campestris pv.  Ref: 9371459 J Bacteriol, 1997
- Based on sequence similarities, the Bordetella pertussis  **tonB**   **exbB**  exbD locus was identified on a cloned DNA fragment.  Ref: 10722583 Infect Immun, 2000
- In gram negative bacteria, high affinity iron uptake requires the  **TonB**   **ExbB**  ExbD envelope complex to release iron chelates from their specific outer membrane receptors into the periplasm.  Ref: 10722583 Infect Immun, 2000
- Sequencing of the Ton system genes revealed that the arrangement of the genes was  **exbB**  exbD  **tonB** .  Ref: 9423852 Infect Immun, 1998
- Protonmotive force,  **ExbB**  and ligand bound FepA drive conformational changes in  **TonB** .  Ref: 10209752 Mol Microbiol, 1999
- Nevertheless, the  **tonB** ,  **exbB**  and exbD1 mutant strains were able to spread systemically in cauliflower.  Ref: 10832632 Microbiology, 2000
- The exbD2 gene as well as the iron uptake genes  **tonB** ,  **exbB**  and exbD1 of Xanthomonas campestris pv.  Ref: 10832632 Microbiology, 2000
- Analysis of sequence alignments of TolQ and TolR homologues from numerous Gram negative bacterial genomes, together with analyses of the different tolQ tolR mutants, revealed that the TM domains of TolQ and TolR present structural and functional homologies not only to  **ExbB**  and ExbD of the  **TonB**  system but also with MotA and MotB of the flagellar motor.  Ref: 11722743 Mol Microbiol, 2001
- In gram negative organisms, high affinity transport of iron substrates requires energy transduction to specific outer membrane receptors by the  **TonB**   **ExbB**  ExbD complex.  Ref: 12562789 J Bacteriol, 2003
- campestris revealed four open reading frames which, based on sequence homologies, were designated  **tonB** ,  **exbB** , exbD1, and exbD2.  Ref: 9371459 J Bacteriol, 1997
- Pasteurella multocida  **exbB** , exbD and  **tonB**  genes are physically linked but independently transcribed.  Ref: 12044675 FEMS Microbiol Lett, 2002
- The  **exbB** , exbD and  **tonB**  genes of the Pasteurella multocida animal pathogen have been cloned by complementation of an Escherichia coli  **tonB**  mutant.  Ref: 12044675 FEMS Microbiol Lett, 2002
- Crystal structures of two outer membrane transport proteins, FhuA and FepA, and biochemical data reveal strong long range conformational changes of the proteins upon binding of Fe 3 siderophore complexes and in response to energy transfer from the cytoplasmic membrane into the outer membrane via the  **TonB**   **ExbB**  ExbD protein complex.  Ref: 11934617 Curr Opin Microbiol, 2002
- Uptake of the mutated colicin U DTMVV depended on  **TonB**  and  **ExbB**  and still remained TolA and TolQ dependent, but no longer required TolB.  Ref: 9682486 FEMS Microbiol Lett, 1998
- PCR approaches allowed the amplification, cloning and sequencing of A. suis  **tonB** ,  **exbB** , exbD, tbpB and tbpA homologues.  Ref: 12742718 Vet Microbiol, 2003
- Reverse transcription RT PCR, using RNA from organisms grown under iron replete and iron restricted conditions, revealed that  **tonB** ,  **exbB** , exbD, tbpB and tbpA are transcribed as a single unit with expression being up regulated in response to iron restriction.  Ref: 12742718 Vet Microbiol, 2003
- **TonB** , the energy transducer, appears to go through a cycle during energy transduction, with the absence of both  **ExbB**  and ExbD creating blocks at two points i in the inability of  **TonB**  to respond to the cytoplasmic membrane proton motive force and ii in the conversion of  **TonB**  from a high affinity outer membrane association to a high affinity cytoplasmic membrane association.  Ref: 12193634 J Bacteriol, 2002
- However, inducible levels of TX 100 resistance in gonococci had an absolute requirement for the  **TonB**   **ExbB**  ExbD system, suggesting that such resistance in gonococci has an energy requirement above and beyond that required for constitutive pump activity.  Ref: 11796379 Antimicrob Agents Chemother, 2002
- The Ton system consisting of the proteins  **TonB** ,  **ExbB**  and ExbD is required for colicin and microcin import.  Ref: 12423780 Biochimie< MedlineTA>Biochimie, 2002
- The ratio of these proteins was calculated at one  **TonB**  two ExbD seven  **ExbB**  under all four growth conditions tested.  Ref: 11967085 Mol Microbiol, 2002
- In Gram negative bacteria, the  **TonB**   **ExbB**  ExbD inner membrane multiprotein complex is required for active transport of diverse molecules through the outer membrane.  Ref: 11737642 Mol Microbiol, 2001
- Allelic replacement of the wild type H. ducreyi  **exbB** , exbD, and  **tonB**  loci with the  **exbB** , exbD, and  **tonB**  deletion resulted in an H. ducreyi isogenic mutant unable to utilize hemoglobin but able to utilize hemin at the same levels as the parent strain to fulfill its heme requirement.  Ref: 9423852 Infect Immun, 1998
- **ExbB**  and ExbD each formed two unique cross linked complexes which were not dependent on the presence of  **TonB** , the outer membrane receptor protein FepA, or the other Exb protein.  Ref: 9811664 J Bacteriol, 1998
- Transport of heme by the ShuA protein requires  **TonB**  and its accessory proteins  **ExbB**  and ExbD.  Ref: 9393841 Infect Immun, 1997
- Vibrio cholerae iron transport haem transport genes are linked to one of two sets of  **tonB** ,  **exbB** , exbD genes.  Ref: 9781885 Mol Microbiol, 1998
- As demonstrated previously,  **TonB**  can undergo at least one conformational change, provided both  **ExbB**  and a functional  **TonB**  transmembrane domain are present.  Ref: 10209752 Mol Microbiol, 1999
- They share homology with the  **ExbB** , ExbD, and  **TonB**  proteins, respectively.  Ref: 11418549 J Bacteriol, 2001
- The energy is transduced to FecA by the inner membrane complex,  **TonB** ,  **ExbB** , and ExbD.  Ref: 12618451 J Bacteriol, 2003
- , Triton X 100 TX 100 did not require the  **TonB** ,  **ExbB** , or ExbD protein.  Ref: 11796379 Antimicrob Agents Chemother, 2002
- The energy of the proton motive force is postulated to be transduced to the transport proteins by a protein complex that consists of the  **TonB** ,  **ExbB** , and ExbD proteins.  Ref: 11566987 J Bacteriol, 2001
- Mutational analysis revealed that the ferric iron uptake genes  **tonB** ,  **exbB**  and exbD1 are necessary for the induction of a hypersensitive response HR on the nonhost plant pepper Capsicum annuum and the induction of typical black rot symptoms on the host plant cauliflower Brassica oleracea.  Ref: 10832632 Microbiology, 2000
- The electrochemical potential of the cytoplasmic membrane CM energizes transport across the outer membrane, which requires an energy transducing device, consisting of the proteins  **TonB** ,  **ExbB**  and ExbD, from the CM to the OM.  Ref: 9377472 Biol Chem, 1997
- It is thought that the proteins  **TonB** ,  **ExbB** , and ExbD function as a coupling device between the cytoplasmic membrane and the outer membrane.  Ref: 9457864 J Bacteriol, 1998
- This gene is clustered with three other genes,  **tonB**   **exbB**  exbD1 exbD2.  Ref: 12646254 Biochem Biophys Res Commun, 2003
- **ExbB**  and ExbD do not function independently in  **TonB**  dependent energy transduction.  Ref: 12193634 J Bacteriol, 2002
- Other biochemical evidence also shows that the proteins that provide energy for iron transport at the outer membrane, such as the  **TonB**   **ExbB**  ExbD system, are structurally very similar across bacterial species.  Ref: 11895294 Curr Top Med Chem, 2001
- Inducible, but not constitutive, resistance of gonococci to hydrophobic agents due to the MtrC MtrD MtrE efflux pump requires  **TonB**   **ExbB**  ExbD proteins.  Ref: 11796379 Antimicrob Agents Chemother, 2002
- Uptake of colicin Js into sensitive bacteria was dependent on the  **ExbB**  protein but not on the E. coli K 12  **TonB**  and TolA, B, and Q proteins.  Ref: 11395459 J Bacteriol, 2001
- In this study, Escherichia coli  **TonB**  was found to be distributed in sucrose density gradients approximately equally between the cytoplasmic membrane and the outer membrane fractions, while two proteins with which it is known to interact,  **ExbB**  and ExbD, as well as the NADH oxidase activity characteristic of the cytoplasmic membrane, were localized in the cytoplasmic membrane fraction.  Ref: 9159515 Mol Microbiol, 1997
- Vibrio cholerae was found to have two sets of genes encoding  **TonB** ,  **ExbB**  and ExbD proteins.  Ref: 9781885 Mol Microbiol, 1998
- When  **ExbB**  and ExbD, as well as their cross talk competent homologues ToIQ and ToIR, were absent,  **TonB**  was found associated with the outer membrane.  Ref: 9159515 Mol Microbiol, 1997
- Quantification of known components of the Escherichia coli  **TonB**  energy transduction system  **TonB** ,  **ExbB** , ExbD and FepA.  Ref: 11967085 Mol Microbiol, 2002
- Analysis of translational fusions to alkaline phosphatase and beta galactosidase confirmed that the  **TonB** ,  **ExbB** , ExbD1, and ExbD2 proteins are anchored in the cytoplasmic membrane.  Ref: 9371459 J Bacteriol, 1997
- Analysis of the nucleotide sequence in the complementing DNA showed that it codes for three genes in the order of  **exbB**  exbD  **tonB** .  Ref: 9074497 Gene, 1997
- campestris  **tonB** ,  **exbB** , and exbD1 are essential for ferric iron uptake, but exbD2 is not.  Ref: 9371459 J Bacteriol, 1997
